# Saphenous and Sciatic Nerve Blockade with and without Obturator Nerve Block for Tibial Plateau Levelling Osteotomy Surgery in Dogs: A Randomized Controlled Trial

**DOI:** 10.3390/ani13243792

**Published:** 2023-12-08

**Authors:** Chiara Di Franco, Chiara Cipollini, Giacomo Figà Talamanca, Giulio Tazioli, Stefano Patroncini, Maurizio Calistri, Angela Briganti

**Affiliations:** 1 Department of Veterinary Sciences, University of Pisa, 56124 Pisa, Italy; chiara.difranco@phd.unipi.it (C.D.F.); chiaracipollini93@gmail.com (C.C.); stefanopatroncini@gmail.com (S.P.); 2 Clinica Veterinaria Valdinievole, Monsummano Terme, 51015 Pistoia, Italy; g.ftalamanca@gmail.com (G.F.T.); taziogiulio@gmail.com (G.T.); maurizio.calistri@gmail.com (M.C.)

**Keywords:** dog, locoregional anaesthesia, obturator nerve, ultrasound, anaesthesia

## Abstract

**Simple Summary:**

Locoregional anaesthesia plays a fundamental role in the correct management of surgical interventions. Veterinary ultrasound-guided techniques are in continuous evolution to obtain adequate analgesia with minimal motor impact. We hypothesized that the block of the obturator nerve in the inguinal compartment, combined with the block of the saphenous and the sciatic nerves, without the block of the femoral nerve, could produce analgesia with less motor function impairment for tibial-plateau-levelling-osteotomy (TPLO) surgery. Having easily identified a window during the anatomical study that included an inguinal approach, we could correctly perform this new ultrasound-guided obturator-nerve-block technique. Patients that received three blocks with ropivacaine had statistically significantly less need for rescue analgesia during surgery than those that received saline solution at the level of the obturator nerve. In the group that did not receive ropivacaine for the obturator nerve block, nociception occurred in almost all patients at the time of incision of the joint capsule, showing the obturator nerve role in the innervation of the knee joint. The use of this new technique in association with the block of the sciatic and saphenous nerves represents an improvement in locoregional techniques in knee surgery, as the patient not only has a reduction in intraoperative nociception but also has better postoperative mobility, resulting from the fact that the femoral nerve, which is responsible for the innervation of the quadriceps muscles, was not blocked.

**Abstract:**

The objective of our study was to compare the efficacy of sciatic and saphenous ultrasound nerve blocks with and without US-guided obturator nerve block in dogs undergoing tibial-plateau-levelling-osteotomy (TPLO) surgery. This study was developed in two phases: identification of an ultrasound window in the inguinal region for obturator nerve block and utilization of it in dogs undergoing TPLO. Dogs were assigned randomly to one of two groups: one received the three blocks with 0.5% ropivacaine (ON group) and the second one (NoON group) with NaCl instead of ropivacaine for the obturator block. In phase 1, the obturator nerve was visible between the pectineus and the adductor muscles and was approached using an in-plane technique. It was possible to use the ultrasound window for phase two. The number of dogs that received at least one bolus of intraoperative rescue analgesia in the NoON group (12/15 dogs) was significantly higher (*p* = 0.003) in comparison with the ON group (4/15). An ultrasound window to block the obturator nerve in the inguinal compartment with an in-plane technique was found. The use of this approach could produce adequate analgesia with less motor function impairment in dogs for TPLO surgery.

## 1. Introduction

Cranial cruciate ligament (CCL) disease is the most common cause of pelvic limb lameness and stifle joint osteoarthritis in dogs. Surgical treatment is frequently recommended for more rapid stifle joint stabilization, meniscal treatment, and an earlier return to clinical function [1]. Tibial plateau levelling osteotomy (TPLO) is one of the most common surgical procedures performed for the correction of this problem [2], and because it involves arthrotomy, osteotomy, and bone plate placement [3], it is considered a quite painful procedure [4,5,6]. In the last few years, several anatomical and clinical studies have been conducted to evaluate the impact of regional anaesthesia techniques in perioperative analgesia for pelvic limb surgery [7,8,9,10,11,12,13,14]. The inclusion of locoregional anaesthesia in balanced anaesthetic protocols has been shown to promote better perioperative outcomes and reduced opioid consumption and morbidity, as well as reduction of the costs [15,16,17,18]. Ultrasound-guided nerve block has been highly developed in the past few years; its potential advantages are many, including direct visualization of nerves and adjacent anatomical structures, direct visualization of the spread of local anaesthetic during injection, and avoidance of intraneural or intravascular injections. 

The lumbosacral plexus is in charge of the innervation of the canine stifle. More specifically, the nerves involved are saphenous, femoral, obturator, and sciatic [19]. In detail, the joint is innervated by the posterior articular nerve and the lateral articular nerve (branches of the sciatic nerve) and by the medial articular nerve, which mainly originates from the saphenous nerve and also contains branches of the femoral (FN) and obturator (ON) nerves. The literature indicates that the blocks of the sciatic and of the femoral nerves are effective for TPLO surgery [7,8,9,10,11,12,13,14]. One of the most important consequences of the femoral nerve block is the motor block of the quadriceps muscle (responsible of the extension of the knee), which may interfere with the ability to walk in the postoperative period. Thus, excluding the femoral nerve from the block and meanwhile including the obturator nerve could be beneficial in terms of motor function and analgesia. Papadopoulos and colleagues reported that the obturator nerve block may have a role if used in combination with proximal sciatic-nerve-blocking techniques to enable unhindered tripodal gait in the immediate postoperative period [20]. Our hypothesis is that the block of the obturator nerve in the inguinal compartment, combined with the block of the saphenous and the sciatic nerves, without the block of the femoral nerve, could produce adequate analgesia with less motor function impairment for TPLO surgery.

The primary aim of this study was to evaluate the intraoperative success rate of the block of the obturator nerve in the inguinal compartment combined with US-guided sciatic nerve (ScN) and saphenous nerve (SfN) blocks in dogs undergoing TPLO surgery. Secondarily, we also aimed to compare the intraoperative and postoperative efficacy of ScN and SfN blocks with and without the obturator block and to evaluate the duration of the postoperative analgesia and motor function of the proposed blocks.

## 2. Materials and Methods

This study was divided into two phases: phase 1, which included anatomical dissection and ultrasound (US) study of the inguinal region to design a PNB, and phase 2, which included clinical application of the PNB designed in phase 1, during TPLO surgery in dogs. This study was approved by the local Committee for animal welfare n° 31/2019. The owners signed an informed consent statement to enrol their dogs in this study.

### 2.1. Phase 1: Anatomical Study

Seven dogs, euthanized for reasons unrelated to this study and without evident musculoskeletal disease, were enrolled. Two fresh cadavers were used for the gross anatomy study, while the other five were frozen after euthanasia and thawed at ambient temperature before the procedure. With the dog lying in dorsal recumbency and the pelvic limb in a neutral position, the inguinal region was dissected on both sides to evaluate the obturator nerve pathway.

Five of seven cadavers (ten pelvic limbs) were used to find the optimal US window to perform the block. With the dogs in dorsal recumbency, a high-frequency 6–15 MHz linear array transducer (Sonosite, SII, FUJIFILM, Italia, S.P.A., Milan, Italy) was placed perpendicular to the pectineus muscle. The objective was to recognize the pectineus muscle and to define the “target zone” to perform the PNB. Then, a volume of 0.1 mL/kg of new methylene blue solution was injected using an in-plane technique [21]. Subsequently, the inguinal region was dissected to confirm the involvement of the nerve. Injection accuracy was checked by evaluating the nerve staining and was considered adequate if a length of ≥2 cm was stained.

### 2.2. Phase 2: Clinical Study

Thirty dogs, undergoing elective unilateral TPLO surgery and ranked by the American Society of Anaesthesiologists (ASA)’s classification system as physical statuses 1 and 2, were enrolled in this study. Before the procedure, physical examination, haematological analysis, and biochemical analysis were carried out. Skin infections, aggressive behaviour, neurological or neuromuscular disease, and owner’s refusal were considered exclusion criteria. Anti-inflammatory drug treatment, if already present, was discontinued 24 h prior to surgery. Food was withheld 10 h prior to surgery, and water access was allowed up to 2 h before premedication. Dogs were randomly divided into 2 groups using online randomization software (random.org (accessed on 2 December 2023)); the group that received the 3 blocks with ropivacaine was called the ON group, and the group in which, for the obturator block, NaCl was used instead of ropivacaine was called the NoON group.

In all dogs, a catheter (20–22 Gauge; Delta Ven 1; Delta Med, Mantova, Italy) was placed in the cephalic vein, and a fluid therapy with Lactated Ringer’s solution at 2–5 mL/kg/h was started and was modulated accordingly during anaesthesia; then, the dogs received a flow-by pre-oxygenation and methadone IV (0.1 mg/kg) (Semfortan, 10 mg/mL, Dechra, Turin, Italy). Approximately 5 min later, anaesthesia was induced with propofol (Proposure; Boehringer Ingelheim Animal Health, Italy S.p.A.) IV, titrated to effect. All dogs were intubated and connected to a rebreathing circuit, and anaesthesia was maintained with isoflurane (Vetflurane, Virbac S.r.l., Milan, Italy) in oxygen throughout an anaesthetic machine (Avance CS^2^ Pro, GE, Milan, Italy). A catheter (22 or 20 Gauge) was placed in the dorsal pedal artery to measure the invasive arterial blood pressure. With all dogs under general anaesthesia, after the limbs were clipped and aseptically scrubbed, US-guided PNBs for obturator, saphenous, and sciatic nerves were performed, for a total of 3 injections for each dog. With the dog in lateral recumbency and the leg of the block uppermost, the ScN block was achieved with the mid-femoral approach described by Echeverry et al. [22]. Then, dogs were put in dorsal recumbency, and the SfN block was performed with the approach described by Shilo et al., 2010 [23], and the ON block was executed using the technique defined in phase 1. For the ON group, a volume of 0.1 mL/kg of 0.5% ropivacaine (Naropina; AstraZeneca, Verona, Italy) was injected for each block, reaching the total volume of 0.3 mL/kg per dog. In the NoON group, the ON block was performed with 0.1 mL/kg of NaCl. The syringes for the blocks were prepared by one clinician not involved in the evaluation of the patients, and the name of the nerve to be blocked was written on each syringe (i.e., ON, ScN, or SfN). The blocks were all performed by clinicians with more than 4 years of ultrasound-guided-block experience; the surgeries were performed by 2 orthopaedics with several years of experience with the technique proposed by Slocum and Slocum [3].

During anaesthesia, heart rate (HR); respiratory rate (fR); systolic, mean, and diastolic (SAP, MAP, and DAP) invasive arterial blood pressure; end-tidal carbon dioxide pressure (PE’CO_2_); end-tidal isoflurane concentration (FE’Iso); and peripheral oxygen saturation (SpO_2_) were continuously monitored with a multiparameter monitor (Avance CS^2^ Pro, GE, Milan, Italy) and recorded every 5 min at specific surgical time points (Table 1) until the end of the procedure. For invasive blood pressure, a precalibrated transducer (Transpac IV Disposable Pressure Transducer; ICU Medical Europe, Rome, Italy), placed at the level of right atrium and zeroed to atmospheric pressure, was used. Intraoperative monitoring started 5 min before the surgery and was recorded as T0. Before the start of the surgery (T0), FE’Iso was set between 1.2% and 1.3%. The absence of the palpebral reflex and the mandibular muscular relaxation were monitored to evaluate the depth of anaesthesia, together with the cardiovascular and respiratory clinical signs. Volume-controlled mechanical ventilation with a tidal volume of 10–15 mL/kg and an fR between 10 and 16 breaths/min was used in all dogs to maintain the PE’CO_2_ within the physiological range (35–45 mmHg). In the case of an abrupt increase in HR, MAP, and/or fR, recorded as an increase of 20% or more in comparison with the previous value [24], a bolus of fentanyl at 1 mcg/kg was administered slowly IV. If the parameters were not restored after two consecutive fentanyl boluses of 1 mcg/kg, an infusion of fentanyl was started at 2 mcg/kg/h and adjusted for each animal based on clinical requirements. As reported in a retrospective study [25], the PNB for each case was considered successful if the total amount of fentanyl administered intraoperatively was <2.1 mcg/kg/h. The number of boluses administered for each dog and the surgical phase in which each was administered were recorded (Table 1); the total amount of intraoperative fentanyl (boluses and infusion) was calculated by dividing the total amount of fentanyl administered by the duration of the surgery (hours) and then by the bodyweight of the patient.

In case MAP was lower than 60 mmHg (7.9 kPa), FE’Iso was reduced by 0.1% if possible. If hypotension persisted, an IV crystalloid bolus (5 mL/kg over 5 min) was administered. Unresponsive hypotension after 1 crystalloid bolus was treated with a dopamine infusion (dopamine hydrochloride, S.A.L.F., Bergamo, Italy), starting at 2 mcg/kg/min and increasing until the MAP was above 60 mmHg. Bradycardia, in which HR < 60 beats/min, associated with hypotension was treated with atropine (atropine sulphate, A.T.I., Bologna, Italy) (0.02 mg/kg IV). After extubation, 2 mg/kg of carprofen (Rimadyl, Zoetis, Rome, Italy) was administered IV. Pain was assessed postoperatively starting 1 h after spontaneous head lifting, using the short-form Glasgow Composite Measure Pain Scale (SF-GCMPS) [26]. The SF-GCMPS was assessed each hour for 10 h after the recovery of the dogs. An SF-GCMPS greater than 5/24 was considered an indication to treat the dogs with methadone (0.2 mg/kg IM) in order not to have extreme pain when the block was not working. Walking ability was evaluated 1 h after the recovery.

### 2.3. Statistical Analysis

With an α error of 0.05 and β error of 0.2, in order to identify a difference of 50% in the number of dogs that will require a bolus of fentanyl as rescue analgesia, the minimum number of dogs necessary for each group was 13; the number was increased to 15 to face eventual losses. A D’Agostino–Pearson test was used to analyse the normal distribution using commercial software (Prism 9; GraphPad Prism Inc., Boston, MA, USA). Parametrical data were expressed as mean ± standard deviation and non-parametrical data were represented as median (range). A chi-square test was used to compare the number of dogs requiring rescue analgesia and rescue treatments for hypotension. An ANOVA test for repeated measures with a post hoc Bonferroni test was used to assess differences for intraoperative clinical parameters in relation to time in each group. A Friedman test with a Dunn’s post hoc test was used to compare pain scores over the time points. Values of *p* < 0.05 were considered significant.

## 3. Results

### 3.1. Phase 1

**Figure 1:**
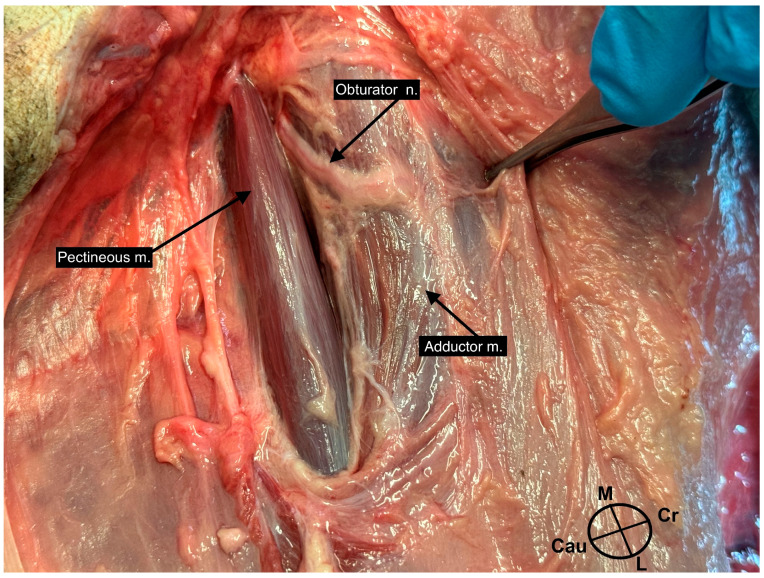
Dissection of the medial aspect of a left leg of 1 of the cadavers enrolled in this study. In the picture, it is possible to identify the obturator nerve emerging from the interfascial plane between the pectineous muscle and the adductor muscle.

The two fresh cadavers used for the gross anatomy study were positioned in dorsal recumbency, and after trichotomy of the entire hindlimb, the skin of the medial portion of the leg was removed and the superficial fascia of the thigh was visible. After the removal of the superficial fascia, in a craniocaudal direction, the sartorius muscle, the femoral artery and vein, the pectineus muscle, the adductor muscle, and the gracilis muscle were distinguished; the ON was visible as a ribbon-like structure emerging between the pectineus and the adductor muscles and moving caudally in a layer between the adductor and the gracilis muscles (Figure 1). Continuing the dissection, the ON was visible running proximally between the pectineus and the adductor muscles. A distal branch directed towards the adductor canal was recognizable only in one leg of the two fresh cadavers.

**Figure 2:**
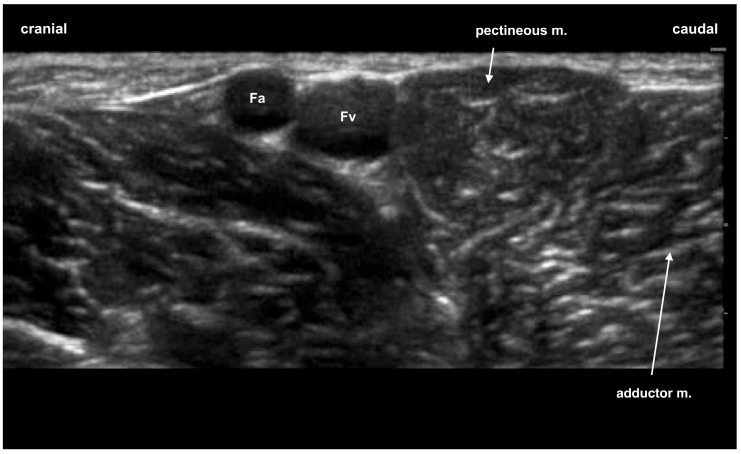
Ultrasound image of the region inspected for the obturator nerve block: the pectineous muscle appears as a heart-shaped structure. Fa, femoral artery; Fv, femoral vein.

**Figure 3:**
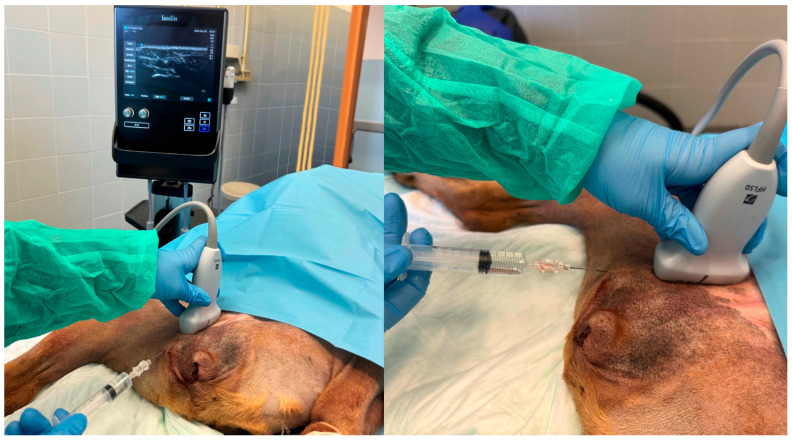
Positioning of the operator, the probe, and the ultrasound machine to perform the block with an in-plane approach, along the visual axis approach. The needle is inserted caudo-cranially, and the probe is positioned parallel to the column over the proximal portion of the pectineous muscle.

**Figure 4:**
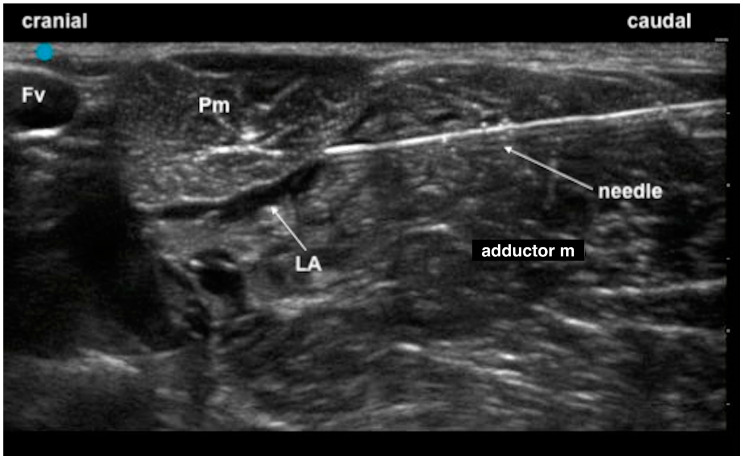
Confirmation of the correct positioning of the local anaesthetic (LA), (the hypoechoic area), inside the interfascial plane between the pectineous muscle (Pm) and the adductor muscle. Fv, femoral vein.

With the dogs in dorsal recumbency and the hindlimb abducted, the probe was positioned on the most proximal portion of the medial aspect of the hindlimb; the pectineous muscle was easily recognizable as a heart-shaped muscle caudal to the femoral artery and vein (Figure 2). After the target was found, the needle was inserted from caudal to cranial, in-plane along the visual axis technique, with the operator positioned on the caudal portion of the leg (Figure 3). The correct injection of the dye was verified by the hydrodissection of the fascial plane of the two muscles (the pectineous and the adductor muscles) (Figure 4, Video S1) in all legs. The dissection showed a complete staining of the obturator nerve inside the interfascial plane for all 10 legs used. No staining of the femoral or the saphenous nerves was highlighted during the dissections.

### 3.2. Phase 2

All thirty dogs enrolled completed this study uneventfully. The mean dose of propofol used for induction was 4.4 ± 1.2 mg/kg, and no differences were found between the two groups. The duration of anaesthesia, duration of surgery, and weight and age of each dog enrolled did not differ between the two groups (Table 2). The number of dogs that received at least one bolus of intraoperative rescue analgesia in the NoON group (12/15 dogs) was significantly higher (*p* = 0.003) in comparison with the ON group (4/15).

The total dose of fentanyl in the two groups was lower than 2.1 mcg/kg/h. Thus, all blocks were considered effective; the ON group received a significantly (*p* = 0.001) lower dose of fentanyl (0.23 ± 0.53 mcg/kg/h) than the NoON group (1.15 ± 0.8 mcg/kg/h). Table 3 summarizes the surgery time points in which rescue analgesia was required for the two groups.

**Scheme 1:**
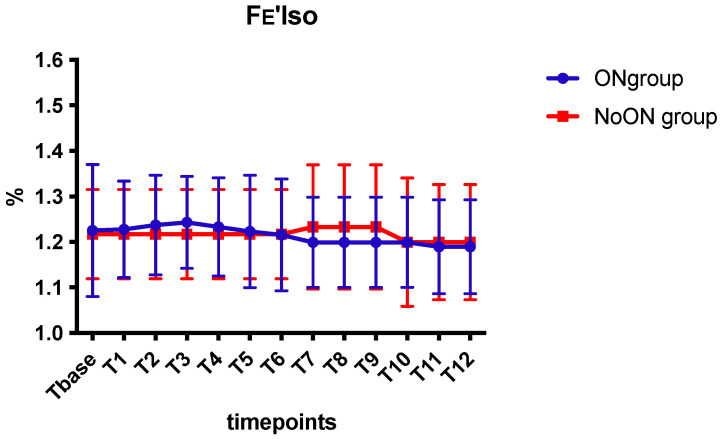
Mean values and standard deviations of FE’Iso in the 2 groups.

No difference was found between the two groups regarding PE’CO_2_, Fe’Iso (Scheme 1), HR, SAP, MAP, and DAP. In the ON group, 3/15 dogs received dopamine infusion, while in the NoON group, 4/15 received it, and no differences were detected. Moreover, no differences were detected in the use of atropine: 2/15 in the ON group and 4/15 in the NoON group.

**Scheme 2:**
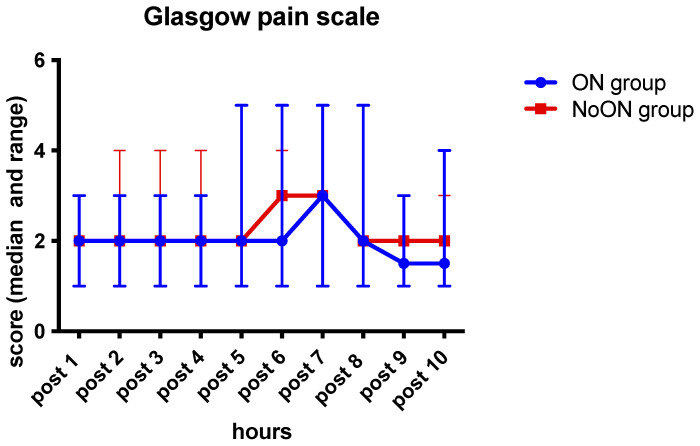
Median and range of the pain score in the 2 groups.

Five dogs for each group required rescue methadone in the first ten hours postoperation. The median pain scores of the two groups are summarized in Scheme 2.

All dogs were able to walk 1 h after the surgery (Video S2). Three dogs with a preoperative lameness of four did not lean on the operated limb even after the surgery, but they were able to walk. Six dogs in the ON group and seven in the NoON group had a proprioception defect (absence of proprioception or retard) (Video S3).

## 4. Discussion

In this study, we demonstrated the perioperative efficacy of a new approach to block the obturator nerve (ON) in the inguinal compartment, combined with the US-guided blocks of sciatic nerve (ScN) and saphenous nerve (SfN), in dogs undergoing TPLO surgery. In phase 1, we confirmed that the ultrasound window reported by Otero and Portela [27] is adequate to block the obturator nerve in the inguinal compartment with an in-plane technique. The technique was found to be easy to perform using the pectineus muscle as a landmark; in fact, it is palpable, and by ultrasound, it is identifiable as a “heart-shaped” muscle. As previously stated, the innervation of the canine stifle is in charge of the femoral, saphenous, sciatic, and obturator nerves [19]. Previous studies reported that sciatic and femoral nerve blocks were sufficient to obtain adequate analgesia in cruciate ligament surgery [7,11,28,29], but the block of the femoral nerve generates a block of the quadriceps muscles, which may have an impact on the walking capacity in the immediate postoperative period, especially if a long-lasting anaesthetic, such as bupivacaine or ropivacaine, is used. The only study present in veterinary literature regarding the selective use of the obturator nerve block during TPLO was conducted by Papadopoulos and colleagues in 2020 [20]. In their study, they associated the obturator nerve block with the femoral and sciatic nerve block, reporting no clinical differences in intraoperative cardiopulmonary values or about the need for rescue analgesia; they therefore concluded that there is not any clinical benefit of an additional obturator nerve block for intraoperative antinociception. Despite this, the authors report a possible advantage in using the obturator nerve block in association with a more proximal approach to the sciatic nerve block in order to facilitate early mobilization in the immediate postoperative period [20]. From the literature, we know that the obturator nerve contributed to the innervation of the joint capsule in 27% of dogs [19]. In our study, only 4 out of 15 dogs in the ON group required rescue analgesia during surgery, whereas 12 out of 15 dogs, approximately 80%, of dogs in the NoON group required it. Given the high percentage, it is possible to assume that the innervation of the obturator is present in a greater portion of dogs than that reported in the literature [19]. It is also interesting that 12 out of 15 patients in the NoON group received a painful stimulus at T3, corresponding to incision joint capsule; this finding confirms that the obturator nerve is implicated in the innervation of the joint capsule, as reported by Huang and colleagues [30]. The ON group received a lower dose of intraoperative fentanyl compared with the NoON group: this further confirms the antinociceptive effect of the obturator nerve block in dogs undergoing TPLO surgery; the reduction in opioid consumption, beyond confirming the analgesic role of the obturator nerve, can also have an economic implication, as already suggested by Warrit and colleagues [17]. In a study conducted in 2021, the authors compared the quality of postoperative analgesia in patients with sciatic and saphenous nerve block, lumbosacral epidural and intraoperative infusion of morphine lidocaine, and ketamine in dogs undergoing TPLO surgery; they reported no significant differences between the groups regarding the duration of anaesthesia [31]. This agrees with the results emerged from our study, where no differences in the duration of anaesthesia were found between the ON group and the NoON group: adding the obturator nerve block does not cause a significant increase in the duration of anaesthesia, probably because the blocks were carried out by skilled clinicians in locoregional techniques. It is therefore to be considered that nerve blocks performed by less experienced individuals may cause variations in anaesthesia time and quality of analgesia [31]. In our study, no significant differences were recorded regarding haemodynamic parameters such as HR, SAP, MAP, and DAP between the two groups: this may be due to the use of locoregional anaesthesia in both groups. Locoregional anaesthesia can reduce complications caused by the systemic administration of drugs such as bradycardia and hypotension [4]. No significant differences regarding FE’Iso were found between the two groups, and the values recorded in our study were comparable to other studies reported in literature [7,20,32]. Concerning postoperative analgesic management, no statistically significant differences were found between the two groups, and all patients in our study were able to walk within 1 h of surgery, both in the ON and NoON groups. As we had hypothesized, excluding the femoral nerve from the locoregional anaesthesia produced adequate analgesia, comparable to sciatic and femoral nerve block, with less motor function impairment. On the other side, patients with grade 4 lameness prior to the operation did not improve their degree of lameness in the immediate postoperative period: this could be due to the patient’s attitude related to the preoperative pain and lameness. This study has some limitations that need highlighting. First of all, the small number of animals is a limitation: this could have affected the real number of dogs requiring rescue analgesia in the NoON group. Secondly, the block was performed by different clinicians, and this may have affected the outcome. Another confounding factor includes differences in the surgical procedure: the operations were performed by two different surgeons, although with similar experience. Furthermore, the protocol involved the administration of an NSAID at the end of anaesthesia; this may have masked the effectiveness of the block postoperatively. However, it was decided by the authors not to modify what is commonly performed in the clinical activity.

## 5. Conclusions

In conclusion, the US-guided blocks of the obturator nerve in the inguinal compartment with an in-plane technique, combined with sciatic nerve and saphenous nerve blocks, produced adequate analgesia with limited motor function impairment and lower need of rescue analgesia in dogs undergoing TPLO surgery. The block of the sciatic and the saphenous nerves alone produced a higher request of intraoperative rescue analgesia but was equally efficacious in the postoperative period.

## Figures and Tables

**Table 1:** Surgery time points registered during the procedure.

Time	Procedure
T0	five minutes before preparation of the operative field
T1	during the preparation of the surgical field
T2	incision skin and subcutaneous tissue
T3	incision joint capsule
T4	distraction of the knee joint
T5	meniscectomy
T6	suture joint capsule
T7	periosteal incision
T8	positioning external fixator
T9	osteotomy
T10	positioning plate osteosynthesis
T11	suture muscle planes
T12	suturing skin and subcutaneous tissue

**Table 2:** Mean values and standard deviation of weight, age, duration of anaesthesia, and surgery in the 2 groups.

	ON Group	NoON Group
Duration of anaesthesia (minutes)	140 ± 20	125 ± 18
Duration of surgery (minutes)	62 ± 12	61 ±13
Weight (kg)	27 ± 10	28 ±11
Age (years)	6.8 ± 3.4	7 ± 3.2

**Table 3:** Number of dogs for each surgery time point that received intraoperative rescue analgesia in the 2 groups.

Time	ON Group	NoON Group
T0	0/15	0/15
T1	0/15	0/15
T2	0/15	0/15
T3	0/15	12/15
T4	2/15	0/15
T5	0/15	0/15
T6	0/15	0/15
T7	0/15	0/15
T8	0/15	0/15
T9	2/15	3/15
T10	0/15	0/15
T11	0/15	0/15
T12	0/15	0/15

## Data Availability

Data supporting the results stated above can be sent to anyone requesting them from the authors.

## Supplementary Materials

The following supporting information can be downloaded at: https://www.mdpi.com/article/10.3390/ani13243792/s1, Video S1: Injection of local anaesthetic in the interfascial plane between the pectineous and the adductor muscles for the obturator nerve block.; Video S2: A dog walking 1 h after the end of the surgery; Video S3: proprioception defect in a dog after the block.
